# Protein Appetite at the Interface between Nutrient Sensing and Physiological Homeostasis

**DOI:** 10.3390/nu13114103

**Published:** 2021-11-16

**Authors:** Md Shahjalal Khan, Redin A. Spann, Heike Münzberg, Sangho Yu, Vance L. Albaugh, Yanlin He, Hans-Rudolf Berthoud, Christopher D. Morrison

**Affiliations:** Pennington Biomedical Research Center, Baton Rouge, LA 70808, USA; shahjalal.khan@pbrc.edu (M.S.K.); Redin.Spann@pbrc.edu (R.A.S.); heike.munzberg@pbrc.edu (H.M.); sangho.yu@pbrc.edu (S.Y.); vance.albaugh@pbrc.edu (V.L.A.); yanlin.he@pbrc.edu (Y.H.); hans.berthoud@pbrc.edu (H.-R.B.)

**Keywords:** amino acids, protein, macronutrient, feeding behavior, homeostasis

## Abstract

Feeding behavior is guided by multiple competing physiological needs, as animals must sense their internal nutritional state and then identify and consume foods that meet nutritional needs. Dietary protein intake is necessary to provide essential amino acids and represents a specific, distinct nutritional need. Consistent with this importance, there is a relatively strong body of literature indicating that protein intake is defended, such that animals sense the restriction of protein and adaptively alter feeding behavior to increase protein intake. Here, we argue that this matching of food consumption with physiological need requires at least two concurrent mechanisms: the first being the detection of internal nutritional need (a protein need state) and the second being the discrimination between foods with differing nutritional compositions. In this review, we outline various mechanisms that could mediate the sensing of need state and the discrimination between protein-rich and protein-poor foods. Finally, we briefly describe how the interaction of these mechanisms might allow an animal to self-select between a complex array of foods to meet nutritional needs and adaptively respond to changes in either the external environment or internal physiological state.

## 1. Introduction: Protein as Unique Nutrient Providing Essential Amino Acids

Animals eat to procure nutrients, with the hallmark example being the need to procure energy (calories). The restriction of energy intake induces a negative energy balance, which triggers a variety of adaptive responses that collectively mitigate the effects of continued energy restriction and serve to rapidly restore energy balance when food becomes available. For instance, energy expenditure is reduced to conserve remaining energy stores, the motivation to find and consume food is increased, and short-term satiety signals (such as gut distension) have a diminished effect, resulting in larger meals once the food is consumed. Many reviews have covered the homeostatic regulation of energy balance, and it is well accepted that a variety of hormones act as nutritional signals and engage neural circuits controlling feeding behavior and energy expenditure [1,2,3,4,5,6,7,8,9,10,11]. However, do animals only defend against energy restriction? For example, it has been widely demonstrated that the depletion of sodium triggers robust physiological (increased sodium retention) and behavioral (increased sodium consumption) adaptations that act to restore sodium balance [12,13,14,15].

Among the three macronutrients, dietary protein is unique in its capacity to provide essential amino acids. Essential amino acids are necessary for survival but cannot be synthesized by mammals and thus are required in the diet. While carbohydrate and lipid act primarily as sources of energy (and essential fatty acids in that case of fat), protein can provide both energy (amino acids can be metabolized) and essential amino acids. It is also a misnomer to think of protein as a single, monolithic macronutrient. Protein is a complex mixture of amino acids, and thus, dietary protein sources can vary in their quality, with high-quality protein sources providing balanced ratios of essential and non-essential amino acids that meet nutritional needs, while low-quality protein sources provide imbalanced ratios often with excess non-essential amino acids. It should be noted that this review’s focus on protein relates primarily to omnivores, which balance intake across a wide variety of food sources. Herbivores (such as a variety of ruminant species) or obligate carnivores (such as cats) exhibit distinct physiological and behavioral adaptations fitted to their unique nutritional requirements [16].

Protein’s role as the only source of essential amino acids naturally leads to the possibility that protein status is monitored or defended, such that animals react both physiologically and behaviorally to the restriction of protein intake and the resultant negative protein balance [17,18,19,20,21]. We and others have written extensively on this topic in recent years, with the consensus being that a variety of species do ‘defend’ protein in a manner that is loosely similar to the defense of energy or sodium [22,23,24,25,26,27,28,29,30,31]. For example, variations in amino acid intake lead to adaptive metabolic changes in the liver, which conserve amino acids during periods of scarcity and metabolize amino acids during periods of excess [32,33,34,35,36,37,38,39,40,41,42]. Thus, the liver serves to buffer against marked changes in circulating amino acids, which is somewhat analogous to its role in minimizing fluctuations in circulating glucose. This important role of the liver in maintaining amino acid balance is relevant when considering that the liver is the primary source of circulating fibroblast growth factor 21 (FGF21; see below). Similarly, variations in dietary protein content also alter feeding behavior, with high-protein diets tending to suppress food intake and low-protein diets tending to increase food intake [28,29,30,31,43,44]. Several labs including our own have demonstrated that rodents on a low-protein diet exhibit spontaneous changes in ‘macronutrient selection’, such that they selectively increase the consumption of high-protein foods while reducing the consumption of food with high carbohydrate or fat [45,46,47,48]. Finally, several studies in humans have also suggested that protein acceptance or preference is increased when total protein intake is low [49,50,51,52,53]. The general observation that low-protein diets drive adaptive changes in food intake or preference is also consistent with the protein leverage hypothesis, which argues that animals prioritize protein intake and as such will overconsume energy to meet protein needs when exposed to low-protein diets [44,54,55]. Observations in a variety of species, including humans, are consistent with this basic observation [56,57,58], although the effect has not been observed universally and varies based on the species and physiological context [44,59,60,61]. 

With this basic introduction, the remainder of this review will focus on mechanisms that might mediate the ability to adaptively shift food preference in response to insufficient protein intake. In particular, this review will focus on the concept that the induction of a protein-specific appetite in response to a protein need state is likely mediated by two discrete but interacting mechanisms, the first linked to long-term signals of protein need, and the second linked to acute meal-related signals that allow the organism to distinguish between foods based on their protein content. 

## 2. Food Choice as an Interaction between Short-Term (within a Meal) and Long-Term (Need State) Signals

How do animals organize behavioral changes in macronutrient preference that are driven by the internal state? We suggest that this response requires two interdependent mechanisms. One mechanism monitors physiological state (i.e., protein balance or protein need state) and signals when an imbalance is present. This classic homeostatic paradigm is expressed in many systems (energy balance, sodium balance) and is typically driven by endocrine signals that act within the brain [1,2,7,8,15,62,63,64]. Currently, the specific mechanisms mediating the long-term detection of protein balance are not clear, but recent work has implicated a variety of possibilities. Regardless of the specific mechanism, it is important to note that this signal of protein need is chronic and ‘meal independent’. In other words, it primarily acts to integrate interoceptive information related to the nutritional state over longer periods both during and between meals. 

In contrast to this long-term mechanism related to physiological need, the act of choosing or discriminating between individual foods is a short-term response that occurs within the meal. In the case of protein preference, animals must identify foods that are high in protein and select them over foods that are low in protein, and this choice is likely mediated by both food cues (taste, smell, sight) and learned associations due to prior experience (food memories). A key contention of this review is that protein choice is likely driven by an interaction between these short-term and long-term mechanisms. When an animal is in a ‘negative protein balance’, long-term signals of protein need to act in the brain to increase protein intake/selection, but they do so by interacting with or gating shorter-term mechanisms used to discriminate between foods. The net effect is that protein-specific cues or foods are incentivized/rewarded/preferred over non-protein, and animals select for and consume more protein. For the remainder of this review, we will highlight available data in support of this overall model, first focusing on long-term mechanisms of protein need before moving to short-term cues related to the protein content within a particular food or meal. However, we acknowledge that in general, there is very little understanding of these mechanisms nor how they might interact within the brain.

## 3. Long-Term Mechanisms Mediating the Detection of a Protein Need State

As described above, dietary protein serves as the sole source of essential amino acids. Consistent with protein’s essential nutritional role, multiple studies demonstrate that chronic protein restriction produces a ‘protein need state’, which is marked by changes in metabolism and feeding behavior. While changes in liver metabolism could be mediated by direct sensing of low amino acid availability, changes in feeding behavior must be driven by changes in the brain. How does the brain detect this protein need state? Below, we discuss several possible mechanisms that could mediate central nervous system (CNS) sensing of protein restriction.

### 3.1. Central Detection of Amino Acid Availability

Since circulating amino acids have access to the brain, the simplest mechanism for the detection of protein need is one in which specific brain areas directly sense fluctuations in circulating amino acids [65,66]. Early work in this area focused on the fact that specific amino acids act as neurotransmitter precursors, and it was hypothesized that tryptophan and tyrosine might represent a protein-specific signal via their role in serotonin and dopamine synthesis [67]. Although variations in protein intake alter the brain tryptophan to amino acid ratio, the administration of tryptophan did not alter protein selection [68,69], and this hypothesis has since fallen out of favor.

More recent studies have focused on the possibility that branched-chain amino acids (BCAA), and particularly leucine, may act directly in the brain as signals of dietary protein [70]. When administered into the brain, leucine suppresses food intake by impacting BCAA metabolism, mammalian target of rapamycin (mTOR), and/or AMPK signaling [71,72,73]. Leucine acts in both the hypothalamus and the brainstem to suppress food intake [71,74], and this effect is not replicated by other amino acids [75]. This important role for leucine is consistent with BCAAs acting as protein signals and their plasma levels increasing in proportion to increasing dietary protein content due to unique metabolic pathways as compared to other amino acids [76]. Importantly, recent work suggests that brain leucine signaling is required for the anorectic effects of high protein diets [74], highlighting the importance of this mechanism for the physiological regulation of feeding. However, it is not clear that brain leucine signaling represents a protein-specific signal or that circulating BCAAs contribute to the detection of a protein need state (low dietary protein). For instance, most work suggests that central leucine acts to broadly suppress food intake, and to our knowledge, no studies have tested whether central leucine injections shift macronutrient preference. While low-protein diets tend to reduce circulating amino acids, circulating BCAAs do not fall extensively in response to low-protein diets, particularly over the longer term [75,77]. In addition, supplementing the diet with BCAAs does not appear to alter protein selection [76], and neither dietary supplementation nor CNS infusion of BCAAs blocks the hyperphagia induced by low-protein diets [75]. Interestingly, recent work by various groups has argued that the restriction of only leucine, isoleucine, all BCAAs, methionine, or the combination of tryptophan and threonine is sufficient to reproduce many of the metabolic effects observed with total protein/amino acid restriction [40,78,79,80,81,82,83]. While these data strongly argue that the dietary restriction of various amino acids triggers a need state, it is not clear if any of these effects are mediated by direct brain amino acid sensing, and indeed, most of these models lead to a robust induction of FGF21 [80,83,84,85].

In summary, a strong body of evidence indicates that amino acids engage specific neural populations controlling food intake. To date, most of this work has focused on models of either high-protein intake or exogenous, brain-specific administration of specific amino acids (leucine), and collectively, these data support the hypothesis that leucine and potentially other amino acids engage distinct nutrient-sensing pathways within the brain to suppress food intake in response to nutrient excess, particularly in response to high protein diets. Contrastingly, there is less evidence to suggest that brain-specific amino acid sensing is essential for adaptive responses to low-protein diets, or that amino acid signaling represents a protein-specific signal. Thus, it is not clear if the brain ‘listens’ to a fall of leucine as a specific signal that would increase the preference for protein.

### 3.2. Gut Hormones as Signals of Protein Status

Signals arising from the gastrointestinal (GI) tract represent another mechanism for a protein-specific signal. High-protein (HP) diets induce a robust suppression of food intake that is greater than an isocaloric load of fat or carbohydrates, and this protein-induced satiety could be mediated by neural (vagal, spinal) or endocrine (gut peptide) signals arising from the GI tract or portal circulation [29,86,87,88]. HP diet stimulates the vagus nerve and induces a pattern of hindbrain c-Fos activation that is distinct from a sucrose load [89], and HP preloads increase glucagon-like peptide-1 (GLP1), peptide YY (PYY), and cholecystokinin (CCK), and glucagon while reducing ghrelin [90,91,92]. In terms of functional studies, vagotomy does not appear to block the anorectic effects of a HP diet [93,94], but at least one study indicates that the anorectic response to a HP diet depends on PYY [95]. Taken together, these data provide substantial support for a model in which high-protein meals suppress food intake by engaging a variety of gut-dependent signals. However, much of this work has focused on the general suppression of food intake as it relates to energy balance regulation, and many of these signals are also altered by fat and carbohydrate loads. We are not aware of any data clearly demonstrating a role for gut peptides in regulating protein preference in response to a protein need state, and thus, it seems likely that the detection of protein need is mediated by other signaling systems. However, as discussed later in this review, it seems possible that some of these GI-derived signals may provide information related to the composition of a meal, and that the brain integrates this ‘within a meal’ information with other signals that are related to the protein need state.

### 3.3. Endocrine Signals of Protein State: FGF21

Downstream tissues associated with amino acid storage or metabolism are another potential source of information related to protein need state, as it would be logical for one or more metabolically active tissues to detect reductions in amino acid availability and produce a signal conveying protein status to the brain. With this model in mind, several years ago, we began work focusing on skeletal muscle and liver as the most likely sources for such a protein-specific signal. This effort culminated in the discovery that the liver-derived hormone fibroblast growth factor 21 (FGF21) is robustly increased in protein-restricted rats, mice, and humans, and that FGF21 is required for mice to sense and respond to protein-restricted states [46,96].

The first studies linking FGF21 to the nutritional state were based on the observation that FGF21 is induced in settings of starvation or ketogenic diets in mice [97,98,99]. Interestingly, these effects did not readily translate to humans [100,101]. However, subsequent work focusing on individual macronutrients demonstrated that FGF21 was induced by high carbohydrate or alcohol consumption [102,103,104,105,106,107,108,109]. Concurrently, work by ourselves and others focused on FGF21 as a signal of protein restriction, with a large number of studies demonstrating that diets low in protein, or select amino acids, produced large increases in both liver FGF21 expression and circulating FGF21 protein levels [39,40,81,83,84,96,110,111,112,113,114,115,116,117,118]. This increase in FGF21 is directly related to low protein intake, as it occurred in settings of both high and low carbohydrate and fat [46,117,119,120,121]. Curiously, in humans, some studies have demonstrated large differences between individuals in their basal FGF21 levels and response to low protein feeding, and the underlying basis for these differences remains unclear [96]. As noted above, the increase in FGF21 is critical for both physiological and behavioral responses to a low-protein diet in rodents, as LP diets alter food intake, energy expenditure, growth, and metabolic endpoints in control mice but not mice lacking FGF21 [39,46,83,96,122,123]. Finally, recent work in our lab indicates that these effects are largely driven by FGF21 action in the brain [46].

In addition to changes in growth and metabolism, more recent work has linked FGF21 to changes in macronutrient preference. This association between FGF21 and macronutrient preference was first established when genetic linkage experiments identified variants in the FGF21 locus that are associated with macronutrient or alcohol intake in humans [109,124,125,126,127,128,129]. It was also demonstrated that exogenous FGF21 treatment suppresses sweet and alcohol preferences by acting in the brain [130,131], providing functional evidence that FGF21 acted to specifically shift preferences between various nutritional sources. This work was followed by the observation that FGF21 acted in the brain to significantly increase protein intake and decrease carbohydrate intake in the classic three-macronutrient choice test [132]. Finally, work in our lab focused on adaptive changes in macronutrient preference in response to the protein-restricted state, and we demonstrated that mice lacking FGF21, or lacking its receptor β-klotho (Klb) in the brain, failed to adaptively shift away from carbohydrate and toward protein in response to protein restriction [46]. Collectively, these data suggest that FGF21 fits the characteristics necessary for a centrally acting endocrine signal of protein need. FGF21 is increased by both low protein and high carbohydrate, acts in the brain to reduce sweet but increase protein intake, and its deletion blocks the adaptive changes in food preference in animals that are protein restricted. However, there are still significant questions related to FGF21′s role as a protein signal. For instance, there remains some uncertainty regarding its primary efficacy in terms of reducing sweet (and alcohol) intake versus increasing protein intake [133], and the mechanisms through which it acts in the brain are largely unclear. Finally, it must be noted that many studies suggest that FGF21 is elevated in settings of obesity, hepatic steatosis, and other settings of metabolic stress that on the surface appear unrelated to a low-protein state [134,135,136], and thus, we lack a unifying framework that explains FGF21′s involvement in these disparate physiological or pathophysiological settings. 

### 3.4. Additional Mechanisms Potentially Related to the Protein Need State

Although the mechanisms described above are all connected to the sensing of amino acid intake or physiological protein need, we acknowledge that additional mechanisms may also contribute to the detection of insufficient protein intake. Perhaps the most intuitive model would be one in which a molecule related to lean body mass or skeletal muscle mass acts as a signal of lean mass in a manner analogous to leptin’s role as a signal of fat mass [137]. Skeletal muscle mass is sensitive to dietary protein content, and skeletal muscle secretes a wide variety of endocrine hormones, myokines, and metabolites that could act on the brain [138]. However, to date, no muscle-derived signal has been clearly linked to protein appetite or the physiological response to protein restriction, although admittedly, there has been very little work in this regard. 

Another endocrine system linked to protein state is the growth hormone (GH)/insulin-like growth factor (IGF-1) axis. GH and IGF-1 play important roles in growth, protein metabolism, and the response to nutrient restriction, and it is well known that IGF-1 levels decrease in response to protein restriction. However, as above, there has been limited work in terms of IGF-1 contributing to protein appetite, and this question is complicated by the fact that IGF-1 acts very broadly to influence many metabolic processes. For instance, the IGF-1 knockout mouse exhibits a very severe phenotype including early postnatal lethality, making it difficult to study their underlying physiology [139]. Interestingly, there is one body of literature that does connect GH/IGF-1 to protein appetite, as it was shown that chronic high-dose GH injections increased protein intake in rats self-selecting between diets that vary in protein content [140]. One interpretation of this observation is that chronic GH drives increased lean mass, resulting in an increased physiological demand for protein and therefore an adaptive shift in protein preference. To date, there have been no studies dissecting the mechanism underlying this effect, specifically whether it involves direct effects of GH on the brain or instead a downstream signal induced in response to the need for protein. It is also not clear if this effect translates to humans [141]. Regardless, this discussion highlights the possibility that additional signals related to growth, muscle mass, or exercise might contribute to protein appetite.

## 4. Short-Term Mechanisms Mediating the Detection and Discrimination of Protein within a Meal

The above section focuses on longer-term, interoceptive cues that communicate information regarding the protein need state to the brain. However, for an animal to selectively increase or decrease the consumption of a specific nutrient in response to physiological need, it must also be able to recognize that nutrient within a specific food. Therefore, this ability to discriminate between foods based on their nutritional composition is a different mechanism from the prior discussion of physiological need.

One method for discriminating between protein-rich vs. protein-poor foods is the direct detection of protein or amino acids within the food. Individual amino acids can be detected by both the gustatory and olfactory systems and are often associated with umami taste, although it is notable that not all amino acids taste similar [142,143,144]. For the gustatory system, taste receptor type 1 members 1 and 3 (T1R1 and T1R3) contribute to both the detection of amino acids and the perception of umami taste, although there is evidence suggesting that additional receptors may also contribute [145,146,147,148,149]. Therefore, although work remains to fully clarify the specific receptors and mechanisms mediating the gustatory detection of various amino acids, it is quite clear that amino acids can be directly and perhaps innately detected by the nose and mouth. In addition, since the critical T1R1/3 receptors are also expressed on some enteroendocrine cells [150,151,152,153], it is also likely that amino acids can be directly sensed within the intestine.

Ingestive behavior is also heavily influenced by learning and memory. That is, a food item with a particular smell or taste is associated with the consequences of intestinal absorption. Then, these learned associations are used to guide future food choices, particularly when there is a need state for a specific nutrient. In a protein-deficient state, an animal would choose food items whose smell and taste (and even other sensory and situational attributes such as environmental cues and context) were previously associated with the absorption of protein/amino acids. On each occasion, such protein/amino acid-specific “food memories” are updated. If the chosen food item contains the expected amount or even more absorbable protein, the memory is strengthened; if it contains less or no protein, the memory is weakened or erased. Although limited, there is evidence to suggest that the selection of protein or amino acids is mediated by such learned behavior. First, rats consuming lysine or threonine-deficient diets did not immediately compensate with increased consumption of the missing amino acid during initial lick-based tests (naive) but did develop preferences after longer-term exposure (experienced) [154], suggesting that rats require post-ingestive learning (conditioning) to discriminate between various amino acid solutions. Similarly, protein-restricted hamsters will exhibit a learned preference for a marker flavor that has been associated with a high-protein food [155], again suggesting that the animals are associating the consumption of protein with specific flavor cues within the food, not necessarily the taste of protein itself.

The main driver of such choice behavior appears to be the association between a particular food cue and the satisfaction or reward gained from absorption of the needed nutrient. While gut signals induced by the absorption of nutrients were traditionally thought to generate satiation and cessation of further intake, it is now recognized that it also comprises an appetitive feed-forward signal, which was coined “appetition” by Sclafani and colleagues [156,157]. These investigators have demonstrated that animals learn to associate the taste and flavor of ingested foods with their post-ingestive consequences [158,159]. By pairing flavored solutions with intragastric or intraduodenal nutrient infusions, animals will manifest a preference for solutions that are paired with the infusion of nutrients [159,160]. Taken together, these data strongly argue that animals find the digestion or absorption of nutrients rewarding, but that the resulting preferences are driven by learned associations with general flavors within the food, not the taste or smell of the specific nutrient per se. Importantly, such post-ingestive conditioning is not unique to protein, and most work in this area has focused on other nutrients, most notably glucose. The initial sensing of the unconditioned glucose stimulus requires the sodium-glucose transporter 1 (SGLT-1) located in the apical membrane of common enterocytes and enteroendocrine cells. Conversely, the unconditioned glucose stimulus does not depend on intracellular glucose metabolism [161,162] and is intact in “tasteless” mice lacking T1R3, gustducin, or transient receptor potential cation channel subfamily M member 5 (TRPM5) [163]. Together, these data suggest that the critical event is therefore the absorption of glucose by cells within the gut.

There has also been significant work focusing on how this GI glucose signal is transmitted to the brain. Based on subdiaphragmatic vagotomies, Sclafani and colleagues did not find a role for vagal afferents in conditioned intestinal nutrient sensing and suggested a humoral mediation of the US signal to the brain [164,165]. In contrast, earlier observations suggested the sensitivity of vagal afferents to a variety of nutrients based on electrical recording from the cervical vagus including glucose, fatty acids, and amino acids [166,167,168,169], and intraduodenal nutrient infusions activated second-order vagal sensory neurons in the nucleus tractus solitarius (NTS) [170,171]. Importantly, some recent observations using modern genetics-based techniques also implicated vagal afferents innervating the small intestinal mucosa in the propagation of the unconditioned glucose signal from the gut to the brain. In one such study, specialized small intestinal enteroendocrine cells (neuropod cells) were shown to penetrate the lamina propria and synaptically oppose vagal afferent endings [172]. Optogenetic silencing of these neuropod cells in mice led to an inability to develop a preference for intestinal glucose versus the non-nutritive sweetener sucralose [173]. In another study, intestinal infusions of glucose and α-methyl-D-glucopyranoside (MDG), but not the non-nutritive sweetener acesulfame K (Ace K), supported preference learning and activated a bilateral subset of proenkephalin-expressing neurons in the caudal NTS (cNTS). These responses were blocked by acute bilateral surgical cervical vagotomy and by the selective silencing of neurochemically-defined vagal sensory neurons in the nodose ganglia [174]. The discrepancy regarding vagal afferent mediation is likely due to the non-selective character of total subdiaphragmatic vagotomies and afferent-selective subdiaphragmatic vagal deafferentation. These crude vagotomies eliminate a great number of vagal fibers with different functionalities and likely lead to adaptive changes in the bidirectional signaling between the gut and the brain over time. Newer genetics-based tools to selectively label and manipulate subpopulations of vagal afferents have recently become available [174,175,176,177,178,179,180,181]. In particular, single-cell RNA sequencing studies may be able to identify more specific markers for vagal sensory afferent neurons that allow the selective manipulation of such specific populations of vagal sensory neurons [177,180,181].

Collectively, the above data demonstrate that animals learn to associate the absorption of glucose with flavor cues. Compared with the intestinal glucose signal, the specifics of intestinal protein/amino acid signaling mechanisms are much less understood. Using intragastric or intraduodenal casein or monosodium glutamate infusions, relatively mild conditioned flavor preferences were demonstrated in both rats and mice [182,183,184,185], and preference learning with monosodium glutamate (MSG) required an intact vagus nerve [185]. Importantly, dietary protein is much more complicated than glucose, given that multiple amino acids could contribute to the generation of conditioning stimulus, and it is currently unknown whether any single amino acid or multiple amino acids convey a protein-specific signal. Overall, we have a very poor understanding of the mechanisms through which protein-specific signals might be detected or signaled to the brain.

## 5. Perspectives and Conclusions

**Figure 1:**
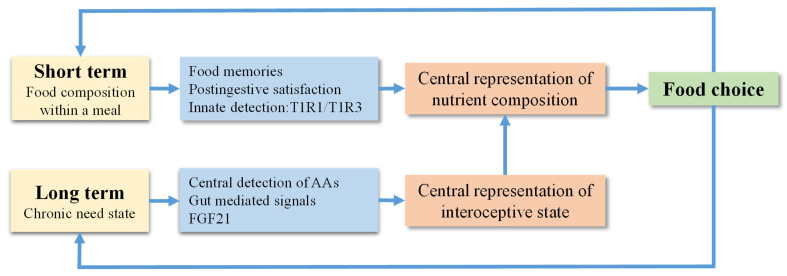
Conceptual mechanism of food choice as an interaction between short-term meal-related cues and long-term signals of the nutritional state. The ability to alter food choice in response to internal nutritional state requires at least two interdependent mechanisms. Within the meal, the animal must sense the nutrient composition of food using sensory cues (visual, gustatory, and/or olfactory inputs), with this information interacting with prior experiences (food memories) to produce a central representation of the nutrient content of a particular food. Separately, the chronic restriction of protein/amino acid availability is signaled via endocrine (possibly FGF21) and/or neural mechanisms, which produce a central representation of interoceptive state. Then, these two central representations interact, such that need state signals shift the value of nutrient-specific cues and/or memories, thereby coordinating adaptive, unconscious shifts in the food preferences that increase the intake of the needed nutrient, in this case protein or essential amino acids.

This review outlines a framework to describe how an animal might increase their consumption of protein in response to a prior period of protein restriction (protein need state). This adaptive response requires two interacting mechanisms (Figure 1). First, the animal must detect this protein need state. Second, it must be able to discriminate between foods and select those that provide protein. If FGF21 is indeed a primary signal of the protein need state and acts to coordinate adaptive shifts in macronutrient preference, then how does FGF21 act to produce these effects? Although it seems clear that FGF21 acts in the brain [46,130,131,186,187,188,189,190,191], our only knowledge regarding the mechanisms through which FGF21 acts to coordinate feeding behavior stems from recent work suggesting that FGF21 acts within the hypothalamus to suppress sweet intake [192]. Similarly, we have very little understanding of how an animal might distinguish between protein-rich and protein-poor foods, although it seems likely that some unique signal associated with the taste and/or post-ingestive consequences of protein or amino acid intake must be generated [193]. Whether this mechanism is analogous to SGLT-1-dependent sensing of post-ingestive glucose is currently unclear. Ultimately, we would suggest that FGF21 must enhance this meal-specific protein signal, such that protein-specific cues that are of low value in the replete state become highly valuable in response to elevations of FGF21 in the protein-restricted state. It is also possible that the value of cues related to non-protein nutrients might be reduced, which is consistent with the strong evidence that FGF21 suppresses sweet intake [130,131].

While it is largely unclear how these two mechanisms might interact to drive food choice, recent work has begun to scratch the surface. One example is the recent evidence that hunger driving agouti-related peptide (AgRP) neurons are acutely inhibited in response to food-related sensory cues but not non-food cues [194,195]. This acute inhibition of AgRP activity precedes the actual ingestion of calories and thus represents a prediction that food ingestion will follow based on known cues. More recent work has argued that this effect is a learning signal that facilitates appetitive behaviors leading to food acquisition [196]. However, this interaction between sensory cues and prior memories likely also involves areas outside the hypothalamus. Indeed, Livneh and colleagues recently focused on a neural circuit including the paraventricular hypothalamus, basolateral amygdala, and the insular cortex that contributes to the integration of both current and future physiological states [197]. The model suggests that both positive and negative motivational state-specific information is gated by visceral and gustatory inputs to the insular cortex to produce behavioral adaptive responses to food cues in the nucleus accumbens, central amygdala, and brainstem areas. As above, this ability to link food cues with the future physiological consequences of ingestion provides a powerful mechanism to explain how ‘food memories’ might drive behaviors that lead to the selection of specific foods. However, it is important to note that these studies have generally focused on feeding as a monolithic behavior; the animal either eats or does not eat. It remains unclear if these recently identified circuits also mediate choices between foods, or whether need-state signals serve to potentiate specific food memories. Finally, considering that animals also balance the need for protein against the need for other essential nutrients, it is unclear how these various need-state cues might interact to guide food intake and preference.

Individuals living with obesity are repeatedly told to make healthy food choices, yet food choice is incredibly complex, often not under conscious control, and we have a poor understanding of the underlying neurobiology. Significant progress has been made in our understanding of the neural circuits regulating total food (calorie) intake and the interaction of palatable foods with neural circuits mediating motivation and reward. However, how the animal balances the needs for protein versus energy, negotiates between nutrient-rich and nutrient-poor foods, and adaptively responds to changes in the internal state remain unclear. We hope that by focusing on the mechanisms mediating macronutrient choice, we can unlock novel neural circuits that mediate preferences between foods and thereby promote healthy choices.
